# Managed Entry Agreements for Pharmaceuticals in the Context of Adaptive Pathways in Europe

**DOI:** 10.3389/fphar.2018.00280

**Published:** 2018-03-27

**Authors:** Jacoline C. Bouvy, Claudine Sapede, Sarah Garner

**Affiliations:** ^1^Science Policy and Research Programme, National Institute for Health and Care Excellence, London, United Kingdom; ^2^Global Pricing and Market Access, F. Hoffman-La Roche, Basel, Switzerland

**Keywords:** adaptive pathways, marketing authorization, drug development, managed entry agreements, Europe

## Abstract

As per the EMA definition, adaptive pathways is a scientific concept for the development of medicines which seeks to facilitate patient access to promising medicines addressing high unmet need through a prospectively planned approach in a sustainable way. This review reports the findings of activities undertaken by the ADAPT-SMART consortium to identify enablers and explore the suitability of managed entry agreements for adaptive pathways products in Europe. We found that during 2006–2016 outcomes-based managed entry agreements were not commonly used for products with a conditional marketing authorization or authorized under exceptional circumstances. The barriers and enablers to develop workable managed entry agreements models for adaptive pathways products were discussed through interviews and a multi-stakeholder workshop with a number of recommendations made in this paper.

## Introduction

**Figure 1:**
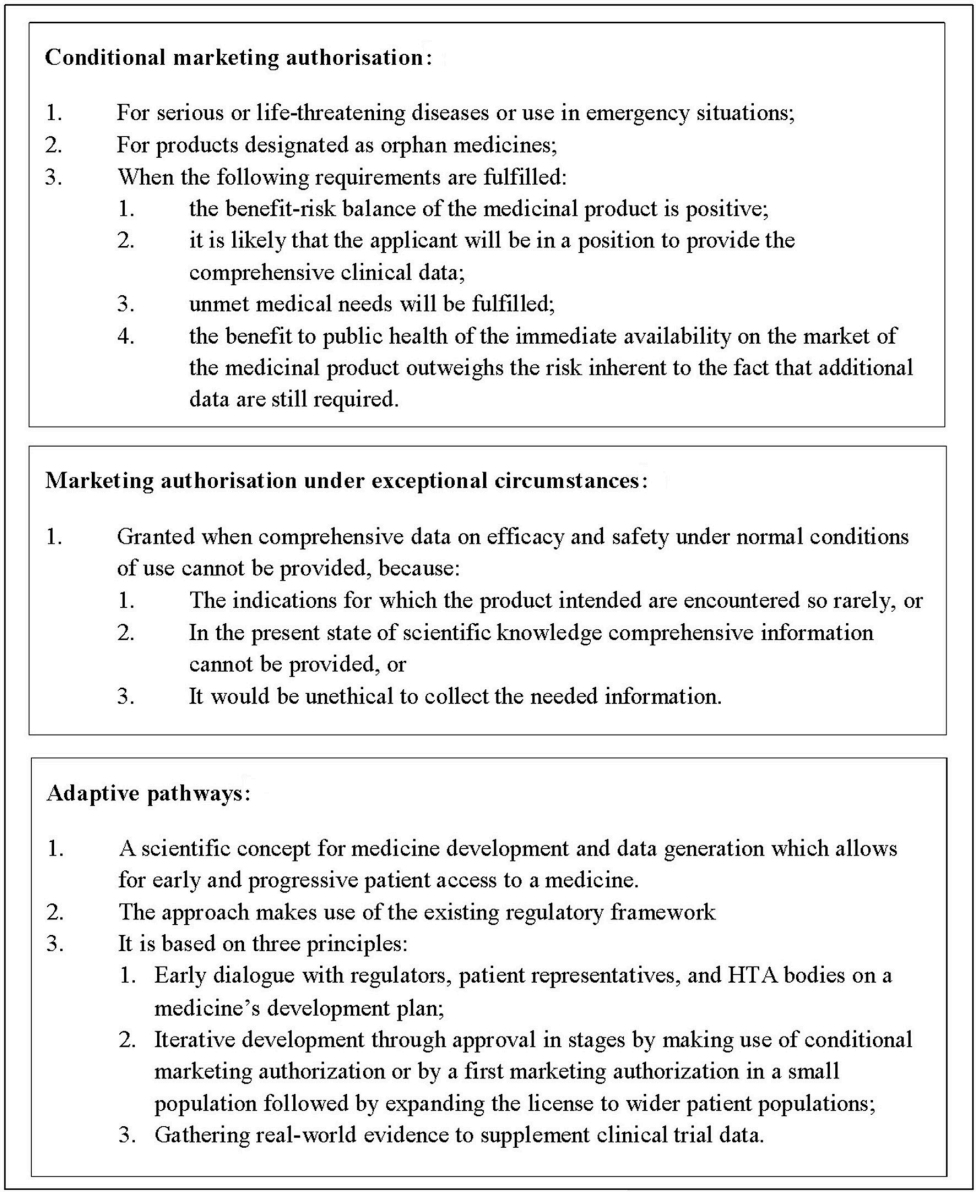
Types of marketing authorization and adaptive pathways for medicinal products in Europe. Adaptive pathways is not a type of marketing authorization but a scientific concept for medicine development and data generation.

The European Medicines Agency (EMA) announced in August 2016 that after a 2-year pilot they would continue the adaptive pathways program as part of their normal procedures. According to Eichler et al. adaptive pathways foresee the managed introduction of a promising pharmaceutical at the earliest appropriate time in the product life-cycle^1^ (Eichler et al., 2012, 2015). In practice, this might mean a (conditional) marketing authorization is granted based on early evidence of the positive benefit-risk profile of the medicine, with a proactive plan for additional evidence generation in place to support the initial license (Figure 1). However, as all products that participated in the EMA pilot are still years away from a (possible) marketing authorization and as the meetings held during the pilot were confidential, it is not yet apparent what the future implications of adaptive pathways for national healthcare systems might be.

In the context of Europe's publicly-funded healthcare systems, the willingness and ability of manufacturers, payers, and health technology assessment (HTA) bodies to find sustainable price and reimbursement solutions to facilitate early patient access is a prerequisite for adaptive pathways to function. This has caused hesitation of especially HTA bodies and payers, as many fear they will end up having to pay for poorly tested yet expensive treatments (Ermisch et al., 2016). In parallel, for manufacturers it is unclear how HTA bodies and payers will assess the initial evidence package submitted for products under adaptive pathways and thus, uncertainty exists regarding possible sustainable price and managed entry models.

Therefore, the Innovative Medicines Initiative (IMI)-funded the ADAPT-SMART Consortium, a public-private partnership that established a multi-stakeholder platform for coordination of adaptive pathways-related activities^2^. One of its work streams was on managed entry agreements for which this paper reports the findings of a number of activities, including a review of the scientific and gray literature, a series of interviews with manufacturers, payers, and HTA bodies, and a multi-stakeholder workshop. Managed entry agreements are contracts that can be used for mitigating the uncertainty regarding a medicine's relative effectiveness, cost-effectiveness, or budget impact. The aim of the activities were to identify enablers and options for suitable managed entry agreements for adaptive pathways products.

## Methodology

**Figure 2:**
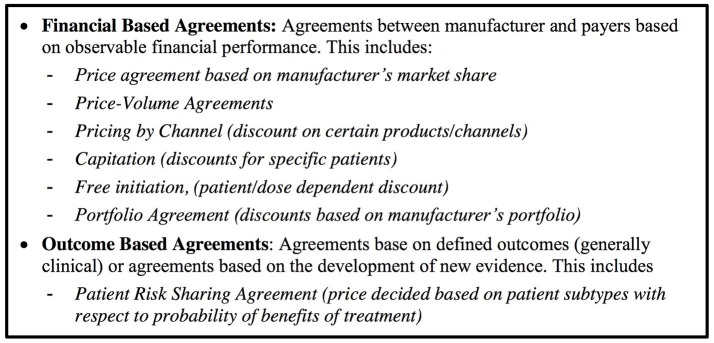
Managed entry agreements—Taxonomy used during the interviews.

**Figure 3:**
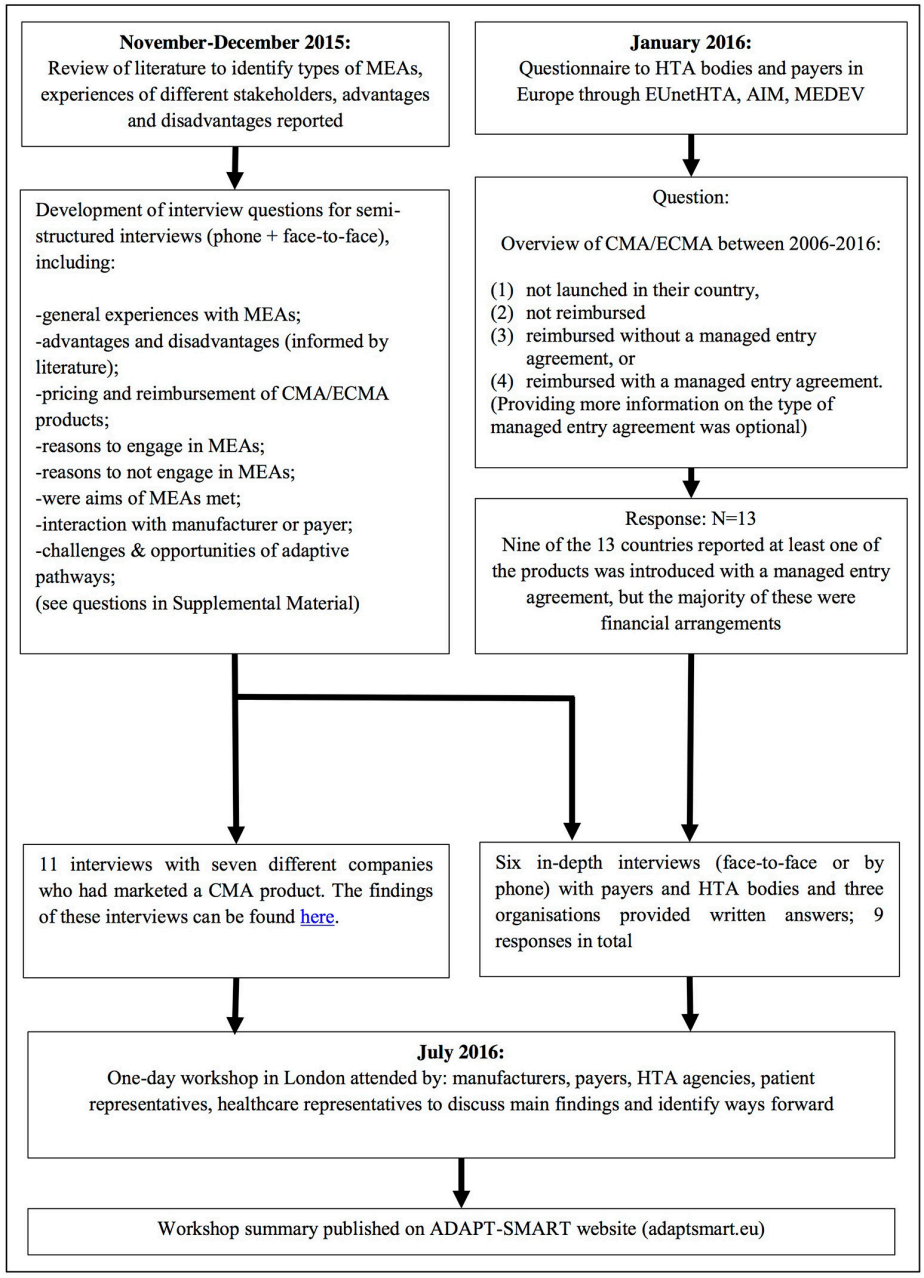
Methods used. MEA, managed entry agreement; EUnetHTA, European network for Health Technology Assessment; AIM, The international Association of Mutual Benefit Societies; MEDEV, medicines evaluation committee; CMA, conditional marketing authorization; ECMA, Exceptional circumstances marketing authorization.

We defined a managed entry agreement as any arrangement between a manufacturer and payer/provider that enables access to a health technology subject to certain conditions (Figure 2). We reviewed the scientific and gray literature on managed entry agreements (Figure 3) using a snowball approach to identify publications that reported (a) stakeholder experiences with different types of managed entry agreements, (b) described different types of managed entry agreements, or (c) reported advantages and disadvantages of their use. The resulting papers were used to develop semi-structured interview questions for interviews with key stakeholders: manufacturers (also marketing authorizations holders), payers, and HTA agencies. In parallel, we sent out a questionnaire to European payers and HTA agencies to ask them about the status of products that had received a conditional marketing authorization or were approved under exceptional circumstances during 2006–2016. As no adaptive pathways product has reached the market (mid 2016), we considered that this subset of marketing authorizations might best reflect the introduction of a product requiring additional evidence generation.

Questionnaire responses were used to identify payers and HTA agencies that had experience with managed entry agreements for these products. In addition, manufacturers who had marketed these products were identified and invited to participate in semi-structured interviews that used the same questions as those used for the payers and HTA agencies interviews. The findings were presented during a 1-day workshop in July 2016. Around 30 people attended the workshop and represented manufacturers, payers, HTA agencies, patient representatives, healthcare representatives, or academics. During the workshop, the findings' implications were discussed and proposals were explored. The following section brings together the results of the review, interviews, and workshop discussions (Figure 1).

## Results

### Financial-based agreements are most common including for products that carry clinical uncertainties

A considerable body of academic work explored the use and theoretical advantages of managed entry agreements for pharmaceutical products (Carlson et al., 2010, 2014; Stafinski et al., 2010; Klemp et al., 2011; Walker et al., 2012; Ferrario and Kanavos, 2013, 2015; Garrison et al., 2013, 2015; Morel et al., 2013; Edlin et al., 2014; Navarria et al., 2015; Faulkner et al., 2016) and the use of financial managed entry agreements (discounts and rebates) is common and widespread in European countries.

The interviews revealed that during 2006–2016 outcomes-based managed entry agreements were not commonly used for pharmaceutical products with a conditional marketing authorization or were authorized under exceptional circumstances in Europe. These products might have been a natural case study for an outcomes-based managed entry agreement as additional data are still required to refine the benefit-risk after initial licensing.

Given that adaptive pathways foresee among other options the use of conditional marketing authorization, this finding has important implications for the introduction of adaptive pathways products and deserves further analysis. It raises the question about the extent to which products approved previously with conditional marketing authorizations would meet the selection criteria for adaptive pathways. It is also possible that not every product that is conditionally approved will automatically have the characteristics that would make it suitable for an outcomes-based agreement. Payers and manufacturers may also have different ideas about what type of product would be a suitable candidate for an outcomes-based agreement.

Hoekman et al. (2015) found that for products with an indication in oncology, conditional marketing authorization tended to be used as a “rescue option” by regulators when the submitted evidence was considered not sufficient to warrant a full marketing authorization (Hoekman et al., 2015). Such a “rescue option” clearly deviates from the pre-planned approach recommended under adaptive pathways. In addition, HTA bodies and payers may have considered that the evidence submitted for regulatory approval of these products was insufficient to appraise the added value of the medicine and thus they may have been reluctant to engage in complex outcomes-based arrangements in this context.

### Outcomes-based arrangements

The interviews indicated that manufacturers seem more interested to explore the use of outcomes-based arrangements than payers. However, there is low appetite among European payers and HTA bodies for using agreements that involve collecting outcomes data due to their complexity.

For a payer, the success of an outcomes-based arrangement ultimately is determined by its ability to reduce the uncertainty regarding the (cost-) effectiveness of a product, reduce its budget impact, improve the efficient use of a product, or a combination of these. Furthermore, reversing a reimbursement decision in light of new evidence needs to be achievable in practice. In the interviews, HTA bodies and payers expressed reluctance regarding outcomes-based agreements because of the added complexity and lack of administrative infrastructure readily available in most countries that would facilitate their implementation (Table 1). In addition, HTA bodies and payers that had experience with outcomes-based agreements reported mixed experiences in the interviews, essentially questioning whether some types of agreements can actually achieve their objectives.

**Table 1:** Summary of main findings from interviews.

**Topic**	**Payers and HTA bodies**	**Medicine developers**
General experience with MEAS	Strong preference from payers and HTA bodies for making a definitive decision regarding the coverage of a new medicine.	Respondents indicated that they had little experience with outcomes-based agreements.
	Payers and HTA bodies prefer financial MEAs to more complicated schemes involving data collection in clinical practice.	Given the payers' concerns primarily revolving around cost and budget impact, financial agreements were much more common and the most straightforward tools.
	Payers questioned whether MEAs really result in meaningful savings for the healthcare system, even when arrangements are purely financial.	Despite the challenges manufacturers indicated in the interviews that they will be willing to explore making use of outcomes based agreements.
Perceived hurdles preventing broader use of outcomes-based MEAs	Increased workload. Monetary streams in healthcare systems (e.g., multiple payers) make tracking of payments challenging and communicating the need for rebates more challenging. Low appetite and difficulty for controlling prescribing in line with label in some countries especially in hospital settings. Lack of infrastructure to collect data in clinical practice. Wide variation in dosing, combination treatments, treatment order, treatment duration make clinical practice data more “messy” and limit comparative effectiveness based on observational data. Increased costs	Resources needed to negotiate and manage such agreements. Some respondents suggested that “once the financial deal is done and a price agreed, there is little interest for payers to engage in additional data analysis to review the price.” Convincing clinicians and hospital pharmacists to conduct administrative tasks to collect data as they see this as an additional burden. Need for real-time individual patient follow-up and measurement of patient data. Lack of experience with outcomes-based schemes in many countries can make the process of negotiation challenging.

*MEA, managed entry agreement; HTA, health technology assessment*.

Furthermore, payers indicated that once a product was made available to patients, delisting it when evidence no longer supported coverage proved impossible due to a lack of mechanisms that would facilitate delisting and low societal support for reversing reimbursement decisions based on economic evidence. These findings have implications for healthcare stakeholders, because if outcomes-based managed entry agreements for adaptive pathways products are needed to facilitate market access, the lack of infrastructure in many countries that would easily enable data collection will need to be addressed and workable models and mechanisms will need to be identified.

Several examples of difficulties with outcomes-based agreements are available in the literature. A Dutch evaluation of an outcomes-based managed entry agreement of oxaliplatin for treatment of stage III colon cancer that collected additional data through a patient registry found that patient heterogeneity made it problematic to estimate incremental cost-effectiveness of the treatment using the collected data (Mohseninejad et al., 2015). A Dutch hematological malignancies registry reported that the three main issues were confounding by indication, missing data, and insufficient comparable patient numbers (Blommestein et al., 2015). In Italy an analysis of 2006–2012 data found that 67% of the expected refunds from certain agreements were actually recovered (Navarria et al., 2015). Reasons identified included difficulty with the required follow-up time, problems with refund notifications, and a lack of (financial) incentive of the prescribing center to participate in a scheme (Garattini et al., 2015; Navarria et al., 2015). These examples highlight the critical importance of robust methods and the clear agreement upfront of stakeholders on the expectations related to the use of the evidence to be collected.

Despite these challenges there are also examples of successful and extensive use of outcomes-based managed entry agreements. Italy has invested in comprehensive systems for data collection on pharmaceutical use in clinical practice which facilitates post-marketing surveillance, and different types of payment models (Pani, 2016). Several payers indicated in the interviews that they were considering or had started to use pay-for-performance models (a sub-type of an outcomes-based managed entry agreement) for some products introduced in recent years. Manufacturers indicated during interviews that they would be willing to explore making use of outcomes-based arrangements as well.

Discussions that took place during the ADAPT-SMART workshop^3^ identified that—apart from technical, healthcare system, and political factors—a lack of trust between payers and manufacturers might be one of the key hurdles to more extensive use of outcomes-based arrangements. Payers and HTA bodies reported that they consistently see very high prices for new products without much differentiation according to added value, whereas manufacturers reported that payers seem more concerned with budget impact and are unwilling to consider more complicated arrangements^4^. We believe that an adaptive pathway might be a well-suited environment to resolve some of these trust issues for the following reasons: (i) adaptive pathways products should have a reasonable expectation of significant added benefit^5^ meaning that questionable product value would be less of an issue, (ii) early dialogue takes place years before products might reach the market which could facilitate the development of feasible payment models if there is broad stakeholder agreement that these products are needed, and (iii) adaptive pathways products would be strictly monitored once on the market, and ways to facilitate monitoring for regulatory and HTA purposes could be explored, for example through the EMA Registries Initiative^6^.

### Regulator vs. payers' evidence requirements

During the interviews manufacturers indicated that they were systematically asked by the EMA to collect additional data through RCTs, safety studies, or patient registries as part of conditional marketing authorizations. However, these studies did not necessarily provide the specific data that national HTA agencies and payers are looking for. Closer alignment of post approval evidence generation requirements between HTA bodies and regulator could mean that improved evidence development could feed into flexible pricing agreements. If there was the possibility to do so, many companies would be keen to explore such opportunities as they could enable value-based pricing.

The perception exists that adaptive pathways products would rely more heavily on observational studies that would replace phase III trials. Yet, in practice a conditional marketing authorization will usually be granted with the legal obligation to perform confirmatory clinical trials rather than solely relying on observational studies. Banzi et al. (2015) reviewed all 24 conditional marketing authorizations between 2006 and 2014 and documented the study designs that were the basis for the approval and the specific obligations requested by regulators (Banzi et al., 2015). Conditional marketing authorizations are usually granted based on either: (a) a single-arm phase II study; (b) a single-arm phase II study plus a phase III RCT (either ongoing or completed); (c) interim-data from a phase III RCT; or (d) double-blinded phase III RCT. Furthermore, in 22 of 24 products the study type the manufacturer was legally obliged to perform to convert the conditional marketing authorization into a full marketing authorization was an RCT; usually, either a phase III RCT, the final analysis of a phase III RCT, or long-term follow-up of a phase III RCT was requested (Banzi et al., 2015). As EMA has stated repeatedly that standards for marketing authorization will not change under adaptive pathways^7^ it follows that RCT evidence is the standard for legal obligations under conditional marketing authorizations rather than observational studies. However, the interviews indicated that despite ongoing evidence development for regulatory purposes, this evidence usually was not utilized by payers or HTA bodies for outcomes-based agreements for these types of products.

### Actionable recommendations from multi-stakeholder workshop discussions

Enabling the earliest appropriate patient access to needed and cost-effective medicines is a primary goal of adaptive pathways and the main motivation for stakeholders to explore the opportunities that adaptive pathways might offer for patients (Bouvy et al., 2016). The multi-stakeholder workshop discussions held in July 2016 made apparent that highest support from all stakeholders exists for products that offer truly transformative potential for patients.

It is expected that under adaptive pathways, companies would engage in a joint scientific advice process involving regulators and HTA bodies which has already happened for some of the products in the pilot. This process should lead to an integrated evidence generation plan, where regulatory requests would be complemented by HTA requests. These requests would be implemented in relevant tools at the national level such as managed entry agreements, in line with national procedures and guidelines as pricing and reimbursement is a national competence. Real-world evidence collected post-initial approval would be used to supplement—not to replace—the original approval requirements and could allow further understanding of optimal product use (e.g., understand patient sub-groups and responders) to maximize effect and minimize risk.

We did not identify existing preferred managed entry agreements that would easily facilitate the managed introduction of an adaptive pathways product. The reasons identified for this through activities reported in this article relate to the way European healthcare systems are organized, the lack of comprehensive data collection infrastructure, and a strong preference of payers to use simpler financial arrangements to manage decision uncertainty. Notwithstanding, multi-stakeholder discussions facilitated through ADAPT-SMART activities reached the following conclusions:

### Multi country data collection

- Coordinating multi-country data collection efforts rather than seeking country-specific models could be facilitated through early dialogue and might facilitate outcomes-based arrangements. If a set of countries could agree on the same data to be collected within their healthcare systems once an adaptive pathways product enters the market this could substantially improve the timeliness, cost, and efficiency of data collection efforts as accrual will be faster. Projects such as the IMI's Big Data for Better Outcomes are aiming to facilitate such coordination efforts^8^.

### Flexible pricing model

- The workshop discussions showed that considering the inherent uncertainty regarding effectiveness, budget impact, and utilization of adaptive pathways products, a flexible pricing model with variable discounts might be acceptable for both payers and manufacturers. Under such a scenario, payer and manufacturer would agree on a list price and conditions under which a discount would be modulated as pre-set outcomes would be met. This way, payers might both have fewer concerns over value for money and manufacturers would be incentivized to ensure that additional evidence is generated. It was however recognized that this model might not be feasible or acceptable in every country. Furthermore, payment models need to be aligned with national processes and tailored to the product in question.

### Enablers of managed entry agreements

- Financial-based agreements are simpler to implement but miss the opportunity to leverage evidence generated post-approval. A possible agreement could combine financial components together with a pre-planned re-assessment taking into account the results of the new evidence generated from RCTs, observational studies, registries, or electronic health records;
- Simple models rather than complicated ones: simple data collection efforts involving the collection of few but essential parameters that are normally tracked by the health care system might be easier to implement, less costly, and more feasible for cross-country coordination;
- Pay-for-performance or risk sharing models were reported to be preferred by several payers rather than conditional reimbursement models or coverage with evidence development models. These models typically involve only paying for patients that experience treatment benefit or not paying for patients who experience early treatment failure;
- Irrespective of the model used, alignment of stakeholder expectations could be achieved by prospectively mapping all potential results of the evidence generation plan as well as clearly defining at the start of the managed entry agreement what the consequences for the price and coverage status of the product will be per the different scenarios.

### Leveraging existing data collection

- The complexity of outcomes-based managed entry agreements could be mitigated by the use of existing data infrastructure to avoid lengthy study set-ups, and the use of simple outcome measures (e.g., time to discontinuation, hospitalization) that are easier to track in clinical practice;
- Properly incentivizing stakeholders (especially prescribers) to participate in data collection could improve data quality and follow-up;
- In the absence of a comprehensive national data collection system, the use of a mutually trusted third party for outcome measurement could facilitate trust;
- Inclusion of data collection efforts in a product's Risk Management Plan (RMP). Although the inclusion of studies that are not requested by regulators cannot be enforced, manufacturers could add the studies to the RMP voluntarily. This might improve the trust of different stakeholders that data collection efforts will be performed as agreed although it would require the Pharmacovigilance Risk Assessment Committee (PRAC) and HTA bodies working together as PRAC may reject proposed studies from the RMP if they are not consider useful to inform the product's benefit-risk profile;
- The survey found that in several countries, many of the conditionally approved products were either never launched or not reimbursed and access to new medicines differs substantially between Member States. More work is needed to determine whether adaptive pathways could cause additional access problems in Member States, especially those with lower incomes.

## Conclusion

At present, many remain concerned about evidence standards for adaptive pathways products (Ermisch et al., 2016; Vella Bonanno et al., 2017). This presents a key challenge that needs to be addressed. Ultimately, patient access to medicines in the European setting is not achieved until products have gone through pricing and reimbursement processes in the different countries. Without the support of HTA bodies and payers in these countries, the uptake of adaptive pathways and workable models for outcomes-based managed entry agreements might remain limited.

## Author contributions

JB and CS: Developed the concept of the paper and the methodology; The first draft was further developed with input from SG. JB and CS: Developed the successive drafts of the paper. All authors read and approved the final manuscript.

### Conflict of interest statement

JB and SG were employed by health authorities at the time of writing this manuscript and CS is an employee of F. Hoffmann La Roche. However, the content of the paper and the conclusions are those of each author and cannot be understood as reflecting those of the organization that employs them. The authors declare that the research has received funding from the Innovative Medicines Initiative 2 Joint Undertaking under grant agreement No 115890. This Joint Undertaking receives support from the European Union's Horizon 2020 research and innovation programme and EFPIA.

## Footnotes

^1^European Medicines Agency Final report on the adaptive pathways pilot. August 2016. Available via http://www.ema.europa.eu/docs/en_GB/document_library/Report/2016/08/WC500211526.pdf

^2^ADAPT-SMART. Accelerated Development of Appropriate Patient Therapies- a Sustainable, Multi-stakeholder Approach from Research to Treatment-outcomes. http://adaptsmart.eu/

^3^IMI ADAPT SMART workshop—Managed entry in the context of adaptive pathways (London, 5th July 2016) Short workshop report. Available via http://adaptsmart.eu/wp-content/uploads/2016/02/ADAPT-SMART-Managed-entry-short-WS-report.pdf

^4^Managed entry agreements in the context of medicines adaptive pathways to patients. Final Report November 2016. Available via http://adaptsmart.eu/wp-content/uploads/2016/12/CRA-MEA-in-the-context-of-MAPPs-Final-Report-16-December-2016-STC.pdf.

^5^IMI ADAPT-SMART. Discussion paper on engagement criteria paper for MAPPs. December 2016. Available via http://adaptsmart.eu/wp-content/uploads/2016/02/ADAPT-SMART-Engagement-Criteria-Final1.pdf

^6^European Medicines Agency. Initiative for patient registries. Strategy and pilot phase. September 2015. Available via http://www.ema.europa.eu/docs/en_GB/document_library/Other/2015/10/WC500195576.pdf.

^7^European Medicines Agency 2016. Final report on the adaptive pathways pilot. August 2016. Available via http://www.ema.europa.eu/docs/en_GB/document_library/Report/2016/08/WC500211526.pdf

^8^IMI Big Data for Better Outcomes. Webpage available via http://www.bd4bo.eu
